# Supplementary material for: A Theoretical and Experimental Study on the Potential Luminescent and Biological Activities of Diaminodicyanoquinodimethane Derivatives
Source: Int J Mol Sci. 2021 Jan 5;22(1):446. doi: 10.3390/ijms22010446 (PMC7795593; doi:10.3390/ijms22010446)
Supplement: Supplementary file 1 [file ijms-22-00446-s001.pdf]

Supplementary Material

A theoretical and experimental approach to diaminodicyanoquinodimethane derivatives with potential luminescent and biological activities.

Edison Rafael Jimenez [1], Manuel Caetano[1], Nelson Santiago[2], F. Javier Torres[3], Thibault Terencio[1, *] and Hortensia Rodríguez [1,*]

[1] School of Chemical Sciences and Engineering, Yachay Tech University. Hda. San José s/n y Proyecto Yachay, 100119, Urcuquí, Ecuador; edison.jimenezg@yachaytech.edu.ec (E.R.J.); mcaetano@yachaytech.edu.ec (M.C.)

[2] School of Biological Sciences and Engineering, Yachay Tech University. Hda. San José s/n y Proyecto Yachay, 100119, Urcuquí, Ecuador; nvispo@yachaytech.edu.ec (N.S.)

[3] Grupo de Química Computacional y Teórica (QCT-USFQ), Instituto de Simulación Computacional (ISC-USFQ), Departamento de Ingeniería Química. Universidad San Francisco de Quito, Diego de Robles y Vía Interoceánica, Quito 17-1200-841, Ecuador.: jtorres@usfq.edu.ec (F.J.T.)

* Correspondence: hmrodriguez@yachaytech.edu.ec ; Tel.: + 593 6 2 999 500 ext. 2622 (H.R.) tthibault@yachaytech.edu.ec (T.T.)

Table of Contents

Table of Contents 1

1.1. Reagents 2

1.2. Equipment 2

2. Chemical section – Synthesis 3

2.1. Synthesis of 1-chloro-4-nitrobenzene (a) 3

2.2. Synthesis of 7-pyrrolidino-7,8,8-tricyanoquinodimethane (b) 3

2.3. Synthesis of N-(4-nitrophenyl)ethylenediamine (c) 3

2.4. Synthesis of N-(2-nitrophenyl)ethylenediamine (d) 4

2.5. Synthesis of 2-(4-(imidazolidin-2-yl)phenyl)malononitrile (1) 4

2.6. Synthesis of 2-(4-(1-(4-nitrophenyl)imidazolidin-2-yl)phenyl)malononitrile (2) 4

2.7. Synthesis of 2-(4-(1-(2-nitrophenyl)imidazolidin-2-yl)phenyl)malononitrile (3) 5

3. AIE studies 5

3.1. Quantum yield (QY) measurements 5

3.2. Theoretical approach 5

3.2.1. Torsion angles 6

3.2.2. Energies of the optimized geometries 7

3.2.3. Mulliken Charges 7

4. Antibacterial Activity 9

4.1. Preparation of inoculums 9

4.1.1. Culture media 9

4.1.2. Bacteria strains 10

4.2. Antibacterial tests 10
4.2.1. Agar diffusion 10
4.2.2. Optical density measurements (OD) 11
5. FT-IR Spectrums 12
6. ESI-MS 12
7. Optimized structures coordinates 13
7.1. Compound 1 13
7.1.1. Ground state S0 13
7.1.2. Excited state S1 14
7.2. Compound 2 15
7.2.1. Ground State S0 15
7.2.2. Excited State S1 15
7.3. Compound 3 16
7.3.1. Ground State S0 16
7.3.2. Excited State S1 17
References 18

1. Materials and Methods

1.1. Reagents

Al reagents were purchased from commercial sources and used without further purification. Acetonitrile were purchased from Scharlau with an HPLC purity grade, 2-chloronitrobenzene, pyrrolidine and ethylenediamine were purchased from Sigma Aldrich with a 99% purity, chlorobenzene, Sulfuric acid were purchased from J.T.Baker with a 97.99% purity, nitric acid was purchased from Emsure at 65% purity, TCNQ, dichloromethane, sodium sulfate. TLC silica gel 60 plates by Merck KGaA were used for thin-layer chromatography. Silica 60 0.04-0.063 mm by Marcherey-Nagel were used for Column chromatography.

1.2. Equipment

UPLC-MS spectrometry was performed on a Waters instrument comprising a binary system manager (ACQUITY UPLC® I-Class) with a reversed-phase column SunFire™ C18 3.5 µm (2.1×100 mm) and an automatic injector and Waters® SYNAPT® G2-S/Si as mass spectrometer. Linear gradients of MeCN (0.01% formic acid) into H_2O (0.01% formic acid) were run at flow rate of 0.3 mL/min. The solvents for UPLC were H_2O (Type I), and MeCN (HPLC quality) with a gradient 5 to 95% MeCN to H_2O. IR spectrometry was performed on a mid-infrared spectrometer (Agilent FTIR, cary 630) equipped with a single reflection, diamond attenuated total reflectance (ATR), working in a wavelength range of 5100-600 cm^{-1}. With a 25 mm interferometer permanently working Michelson 45°. UV-Vis spectrometry was performed on a double beam, double monochromator ratio recording UV/Vis/NIR, (PerkinElmer Lambda 1050). Equipped with Tungsten-halogen and deuterium lamps operating from 175-3300 nm with a detector photomultiplier R6872 for high energy in the UV/Vis wavelength range, controlled by PerkinElmer WinLab software. The fluorescence spectrums were measured with a miniature Fiber Optic Spectrometer, 2-MHz analog-to-digital (A/D) converter, 2048-element CCD-array

detector, resolution to 0.1 nm (FWHM), (USB2000+, Ocean Optics), connected to a PC via USB 2.0 port controlled by OceanView software. The source used for excitation was a Fabry-Perot laser diode class 3R, 405 nm, bandwidth 1 nm, output power 4.0 mW, vertically polarized and multi-longitudinal mode, collimating lens (Thorlabs, model LDM405). Thermo-scientific Nanodrop UV-Vis spectrophotometer.

1. Chemical section – Synthesis

1.1. Synthesis of 1-chloro-4-nitrobenzene (a)

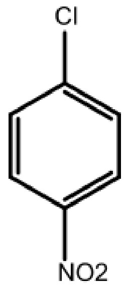

a

Procedure reported in Ref.[1] was followed with minor modifications. Chlorobenzene (112 g, 1 mol, 1.45 eq) is added to a round-bottom flask and heated on a water bath to 40°C. With a vigorous stirring a mixture of sulfuric acid (60 g, 32.6 ml, 0.95 mol, 1.38 eq) and nitric acid (68 g, 45 ml, 0.69 mol, 1 eq) is added dropwise to the chlorobenzene. Addition of the acid requires around 10 minutes, controlling that the temperature is between 40 – 50 °C. Stirring is continued for two hours while the temperature is slowly reduced. Then, the mixture gets cooled in an ice-bath, occasionally stirred with a glass rod until the product precipitate. After standing in the ice-bath for some time, precipitate is filtered off and washed with cold water, to yield p-chloronitrobenzene **a**. The precipitate has an appearance of light-yellow flakes (80 g, yield of 50.77 %).
Mp: 79.9-81 °C (Lit.[2] 80-83 °C)

1.2. Synthesis of 7-pyrrolidino-7,8,8-tricyanoquinodimethane (b)

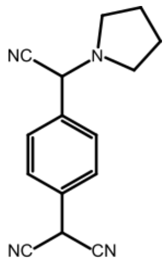

b

Procedure reported in ref.[3] was followed with minor modifications. Pyrrolidine (69.7 mg, 81µl, 0.98 mmol; 0.8 eq) was added at once to a warm solution (50 °C) of 7,7,8,8-tetracyanoquinodimethane (TCNQ) (251 mg, 1.22 mmol, 1 eq) in 20ml of acetonitrile. Initially the TCNQ solution presents a green color which, after the addition of pyrrolidine, changes to a dark green for a split second and rapidly turned dark purple. The solution was stirred at 60 °C for 4.5 hours. After this, the solution was cooled to room temperature and then stored in the fridge for three days to allow the crystallization of the product. Finally, the mixture is filtered out to obtain fine purple crystalline needles, which are washed with cool acetonitrile (3x5 ml) and dried over a day under reduced pressure to obtain **b** (199.3 mg, yield of 80.36 %).
Mp: decompose at 190 °C
FT-IR (ATR) ṽ (cm-1): 2192.44(w), 2163.23(w)
UV/Vis (ACN) λ_{max} (nm): 559
ESI-MS (m/z): calculated for $C_{15}N_4H_{12}$ = 248.3255, found: 249.0986 corresponding to $[M\text{-}H]^-$

1.3. Synthesis of N-(4-nitrophenyl)ethylenediamine (c)

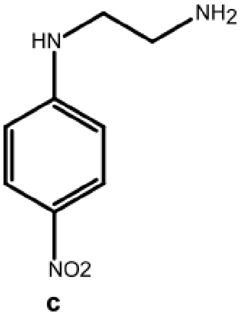

c

Procedure reported in Ref [4] was followed with some modifications. Previously synthesized 1-chloro-4-nitrobenzene a (0.79 g, 5 mmol, 1 eq) was dissolved in a round bottom flask with ethylenediamine (1.5 g, 1.67 ml, 25 mmol, 5 eq). Then, the reaction temperature was raised to 110 – 120 °C keeping a vigorous stirring for six hours. After that, the mixture cooled at room temperature, and the evaporated under reduced pressure. The crude is dissolved in the minimum amount of ethanol and purified by chromatographic column, using methanol as mobile phase. All fractions were analyzed by TLC and the matching phases were collected and

concentrated under reduced pressure to obtain c as light orange crystals (0.404 g, yield of 44.64 %)
Mp: 144-145 °C (Lit.[5] 148-150 °C)
UV/Vis (ACN) λ_{max} (nm): 382

1.4. Synthesis of N-(2-nitrophenyl)ethylenediamine (d)

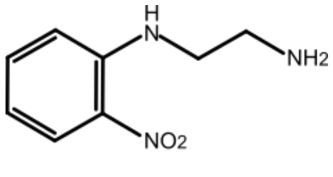

d

Procedure reported in Ref [6] was followed with some modifications. Comercial 1-chloro-2-nitrobenzene (1.58g, 10mmol, 1eq) was dissolved in a round bottom flask with ethylenediamine (2.97 g, 3.3 ml, 50 mmol, 5 eq). Then, the reaction temperature was raised to 110 – 120 °C keeping vigorous stirring for five hours. Later, the mixture was cooled to room temperature, and then evaporated under reduced pressure. The crude was re-dissolved in DCM and dried with Na_2SO_4. Finally, the product was filter and concentrated under reduced pressure to obtain **d** as light orange crystals (1.52 g, yield of 83.97 %).
Mp: 256-258 °C (Lit.[6] 260-261 °C)

1.5. Synthesis of 2-(4-(imidazolidin-2-yl)phenyl)malononitrile (1)

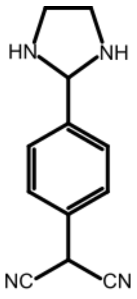

1

Procedure reported in Ref [3] was followed with minor modifications. Ethylenediamine (26.6 mg, 0.44 mmol, 1 eq) was added to a previously warmed solution (at 40 °C) of **b** (110 mg, 0.44 mmol, 1 eq) in acetonitrile (10 ml). The solution changed rapidly from deep purple to an intense green and within the next minutes it changed to yellow. Then, the temperature was raised to 70 °C and the reaction was stirred over the next four hours. The mixture was cooled slowly at room temperature for three hours and let it rest overnight in the fridge to allow the product crystallization. Finally, the mixture was filtered off and washed with cool acetonitrile (3x5 ml) to yield a fine grain yellow powder (66.3 mg, yield of 71.75 %).
Mp: Decompose at 317 °C (Lit.[7] Irreversibly decompose at 280 °C)
FT-IR (ATR) ṽ (cm-1): 3178.23(m), 2183.97(m), 2144.11(s), 1578.25(vs), 1503.04(vs), 1325.32(vs)
UV/Vis (ACN) λ_{max} nm: 408
ESI-MS (m/z): calculated for $C_{12}N_4H_{10}$ = 210.2332, found: 210.1280 corresponding to $[M+H]^+$

1.6. Synthesis of 2-(4-(1-(4-nitrophenyl)imidazolidin-2-yl)phenyl)malononitrile (2)

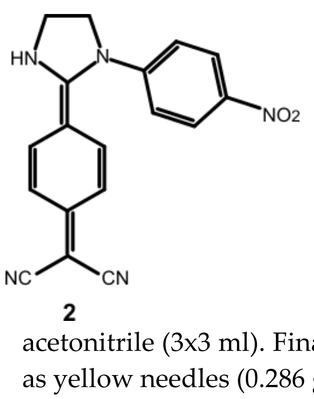

2

Procedure reported in Ref [3] was employed as an analogous method for the synthesis of 2-(4-(1-(4-nitrophenyl)imidazolidin-2-yl)phenyl)malononitrile **2**. Compound **c** (0.3 mg, 1.66 mmol, 1 eq) was dissolved in 10 ml of acetonitrile and added to warm solution (at 50 °C) of PTCNQ **b** (0.411 g, 1.66 mmol, 1 eq) in 10 ml of acetonitrile. The solution was heated and stirred to reflux for seven hours and then it was allowed to cool down at room temperature and immediately after it was stored in fridge for one day to allow the product crystallization. The obtained precipitate was filtered in vacuum and washed with cool acetonitrile (3x3 ml). Finally, the obtained solid is recrystallized in acetonitrile to get product **2** as yellow needles (0.286 g, yield of 52.15 %).
Mp: 161.9 – 163.7 °C
FT-IR (ATR) ṽ (cm-1): 3282.74(m), 2175.03(m), 2123.65(s), 1593.89(vs), 1547.23(m), 1448.98(m), 1305.40(vs)
UV/Vis (ACN) λ_{max} (nm): 371
ESI-MS (m/z): calculated for $C_{18}O_2N_5H_{12}$ = 331.3255, found: 331.1743 corresponding to $[M+H]^+$

1.7. Synthesis of 2-(4-(1-(2-nitrophenyl)imidazolidin-2-yl)phenyl)malononitrile (3)

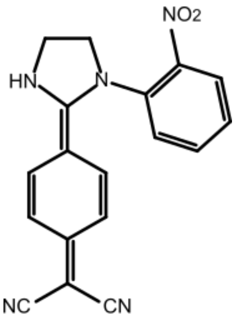

3

A similar procedure as before was employed as an analogous method for the synthesis of 2-(4-(1-(2-nitrophenyl)imidazolidin-2-yl)phenyl)malononitrile **3**. PTCNQ **b** (101.3 mg, 0.4 mmol, 1 eq) was dissolved in 10 ml of acetonitrile at 40 °C. Then, compound **d** (73.84 mg, 0.4 mmol, 1 eq) was added to solution. The reaction was heated at 60 °C and let it stir over four days. The reaction was followed by thin layer chromatography, which showed that after four days the reaction does not go further.

2. AIE studies

2.1. Quantum yield (QY) measurements

Quantum yield measurements (QY) were performed using the comparative method of Williams et al,[8] which involves the use of characterized standard samples with previously known QY values. With this approach, it can be assumed that standard and test sample solutions with absorbance at similar wavelengths will be absorbing the same number of photons. Therefore, a ratio between the integrated fluorescence intensities of the two solutions (recorded under identical conditions) will yield the ratio of the quantum yield values. Since QY is known for the standard sample, the QY for the test sample can be easily calculated.[9]

Hence, for this approach compound **1** was used as the standard sample and compound **2** as the test sample. Both were diluted in DMSO at a concentration of 200 µg/ml and placed in scrupulously clean 1cm fluorescence cuvettes. The emission spectra of both, reference and sample were recorded in full and corrected for the blank (solvent only) to perform proper emission intensity integration. The configuration used for data collection was a 180° excitation-fluorescence detection geometry, with a slight deflection of the optical fiber from the excitation source to avoid any interference signal from the source. Additionally, a careful description of the fluorescence spectrum must be done by the selection of a mathematical function that adequately describes the profile of the spectrum. Due to the considerable asymmetry exhibited by Fluorescence spectra, Log-Normal profiles are most often used.[10] Also, the absorption (optical density) of both samples were recorded under the same conditions as the emission spectrum. Finally, the relative quantum yield (QY) of the sample in the solvent used (DMSO) was calculated according to in **equation (1)**

$$\Phi_x = \Phi_{st} \frac{Int_x}{Int_{st}} \left(\frac{1-10^{A_{st}}}{1-10^{A_x}}\right) \frac{n_x^2}{n_{st}^2} \quad (1)$$

Where, **Φ** refers to the quantum yield, **Int** is the area under the emission peak, **A** states for the absorbance at the excitation wavelength and **n** is the refractive index of the used solvent. The subscripts **st** and **x** denotes the values of the standard and test samples, respectively.

2.2. Theoretical approach

Quantum chemical calculations has been conducted in DADQ derivatives to obtain a further insight into the possible reasons of fluorescence enhancement produced in these molecules. All

geometry optimizations were initially optimized using a force field approach with Avogadro and later with the program package Gaussian 16 (revision A.03). The ground and excited state optimizations were carried out at the Cam-B3LYP/def2TZVP level of Density Functional Theory (DFT), adding GD3BJ as the dispersion correction factor.[7] In addition, the Polarizable Continuum Model (PCM) using the integral equation formalism variant (IEFPCM) as the SCRF with acetonitrile (ACN) was used as implicit solvent model. The structure optimizations were ensured to be at its minimum through frequency calculation in each structure.

2.2.1. Torsion angles

It is important to remark that either the quenching or enhancement of the DADQs fluorescence is directly related and mainly controlled by the photoinduced intramolecular torsions produced at each of the dihedral angles D_γ, D_δ for **1** and D_α, D_β, D_γ, D_δ for **2** and **3**. A further insight into these geometrical changes is required. Thus, it is necessary to measure the dihedral angles at each of the possible torsion sites (**Figure S1)**.

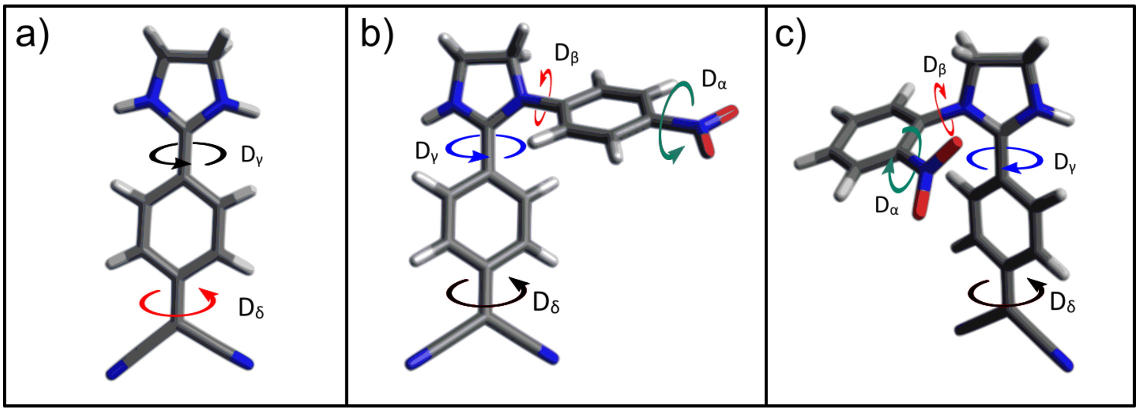

Figure S1. Kinds of intramolecular torsion angles that affect the fluorescence deactivation mechanism in DADQs: a) 2-(4-(imidazolidin-2-yl)phenyl)malononitrile **(1)** with the two types of dihedral angles D_γ, D_δ. b) 2-(4-(1-(4-nitrophenyl)imidazolidin-2-yl)phenyl)malononitrile **(2)** with the four types of dihedral angles D_α, D_β, D_γ, D_δ. c) 2-(4-(1-(2-nitrophenyl)imidazolidin-2-yl)phenyl)malononitrile **(3)** with the four types of dihedral angles D_α, D_β, D_γ, D_δ

The measurements of the dihedral angles in each of the molecules are shown in **(Table S1)**, as we can see these values are quite variable in some cases and remain mostly the same in other cases. The D_δ dihedral angle remains mostly unchanged not only between excited and ground states but also between all three compounds, this tells that the bottom section of the molecule remains mainly unaltered by conformational or configurational changes produced in the upper section of the molecule.

**Dihedral angles**	**1 (S_0)**	**1 (S_1)**	**2 (S_0)**	**2 (S_1)**	**3 (S_0)**	**3 (S_1)**
D_α	-	-	1,5°	1,9°	34,4°	22,9°
D_β	-	-	-40,4°	-20,7°	53,3°	43,3°
D_γ	16°	5,8°	-33,7°	-20,5°	31,4°	38,5°
D_δ	- 0,3°	0,4°	0,2°	0,3°	-0,4°	0,4°

Table S1. Dihedral angles of compounds **(1), (2)** and **(3)** at the ground **(S_0)** and excited **(S_1)** states.

In the case of D_γ a completely different behavior is observed (**Table S1**), there is a variation of around 10° between the excited and the ground state in all three compounds. Additionally, it is a great variation between **1**, **2** and **3** derivatives, this shows that substitutions in the imidazolidine like moiety induces a change in this dihedral angles.

For D_β there are important rotations produced either by the different substitutions in each compound and by the change of the electron disposition in the S_1 and S_0 states. Furthermore, it seems to produce a loss in electrons delocalization around the Quinone section of **3** due to a loss of planarity in the molecules excited state.

Finally, as we can see in **(Table S1)** for D_α the rotation is mainly negligible for **2**, which changes only 0,4° between the excited and the ground states, showing that the electrons remain mostly delocalized between the aromatic ring and the nitro group. On the other hand, in **3** there is a completely different story. The nitro group is strongly deviated from the aromatic ring either in the ground state and excited state, this may be due to the strong steric hindrance produced by the presence of the nitro group in the orto-position. Because of this, the electronic conjugation between the benzene ring and the nitro group gets highly reduced.

2.2.2. Energies of the optimized geometries

With respect to the energies associated to the optimized ground and excited states, in **(Table S2)** are specified the calculated energies for each of the compounds and the energy variation between S_0 and S_1 states. Additionally, in **(Table S3)** are presented the energies associated with compounds **2** and **3** and the difference in energy between those two compounds. The calculated energies show that compounds **1** and **2** slightly change from the ground to the excited state, whereas for **3** the change of energy between the two states is 4 and 3 times larger than compounds **1** and **2**, respectively. This high differences in energy between the states S_0 and S_1 in compound **3** shows a very different excited state that may cause a lack of fluorescence in this molecule.

		**1**	**2**	**3**
Energy (hartree)	S_0	-682,215573965	-1117,75295526	-1117,74682374
	S_1	-682,210919905	-1117,74558489	-1117,72531889
Energy change (KJ/mol)	ΔE	12,2169075	19,34722125	56,45023125

Table S2. Energies and their variation in the optimized geometries of 2-(4-(imidazolidin-2-yl)phenyl)malononitrile **(1),** 2-(4-(1-(4-nitrophenyl)imidazolidin-2-yl)phenyl)malononitrile **(2)** and 2-(4-(1-(2-nitrophenyl)imidazolidin-2-yl)phenyl)malononitrile **(3)** in their ground (S_0) and excited state (S_1).

In addition, the difference between the energies of **2** and **3** indicates that **2** is more stable than **3** in ground state, but this stability is much more noticeable between the excited states of both compounds. (**Table S3)**

	**Energy (hartree)**		**Energy change (KJ/mol)**
	**2**	**3**	ΔE
S_0	-1117,75295526	-1117,74682374	16,15962096
S_1	-1117,74558489	-1117,72531889	53,411043

Table S3. diference of energies between the ground and excited states of 2-(4-(1-(4-nitrophenyl)imidazolidin-2-yl)phenyl)malononitrile **(2)** and 2-(4-(1-(2-nitrophenyl)imidazolidin-2-yl)phenyl)malononitrile **(3)**.

1.2.1. Mulliken Charges

About the distribution of charges in **1**, **2** and **3** we can see that the conformation and the configuration of the molecules does not affect the bottom part of the molecule (dicyanomethane and quinone moieties). However, in nitrogen atom N4 shown **(Figure S2),** the charge strongly decreases from **1** in comparison with **2** and **3. (Table S4)** The increase of negative charge value in **1** is due to the absences of nitrobenzene moiety, present as *N*-substituent for **2** and **3** derivatives. The nitrobenzene scaffold promotes the mentioned behavior due to the electron withdrawing effect of nitro group.

Regarding **2** and **3** derivatives, it is noticeable that the carbon atom directly bonded to the nitro group will be strongly affected by its presence. In the case of **3**, C18 showed a positive charge of +0,11 while for **3** the charge in C18 was the opposite -0,11. The same behavior is expected for C14, which for **2** showed a charge of -0,14, while for **c** it has a +0,08 charge (**Table S4**).

Additionally, the same calculations were performed for p-nitrotoluene and o-nitrotoluene to compare the effect of the different nitro substitutions in the aromatic ring. Whereas, we can observe that the orto-substitution has a slightly higher negative charge located into the oxygens of the nitro group than the one´s in para-substitution. In **2** and **3** there is an opposite behavior, showing that para-substitution has a higher negative charge at the oxygens of the nitro group than the orto-substitution. These results could be explained by the loss of planarity between the nitro group and the aromatic ring in **3**. This loss of planarity reduces the inductive capacity of the nitro group, preventing the adequate attraction of the electronic density from the aromatic ring to the nitro group.

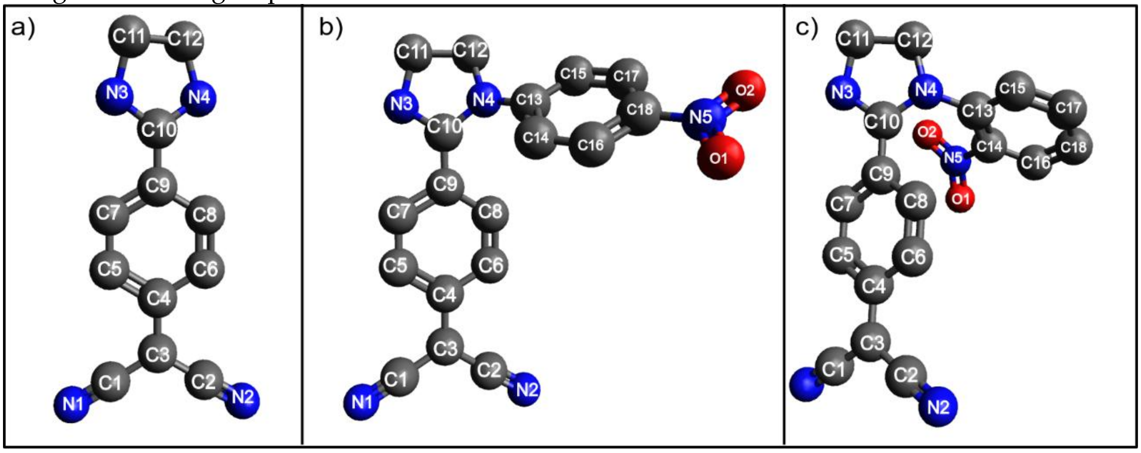

Figure S2. Labeled atoms on 2-(4-(imidazolidin-2-yl)phenyl)malononitrile **(a),** 2-(4-(1-(4-nitrophenyl)imidazolidin-2-yl)phenyl)malononitrile **(b)** and 2-(4-(1-(2-nitrophenyl)imidazolidin-2-yl)phenyl)malononitrile **(c)**.

	**1**		**2**		**3**	
Mulliken charges S_0	C1	-0,257120	C1	-0,257330	C1	-0,257592
	C2	-0,257144	C2	-0,256311	C2	-0,256042
	C3	-0,030085	C3	-0,030837	C3	-0,030362
	C4	0,163865	C4	0,165590	C4	0,172198
	C5	-0,277889	C5	-0,274670	C5	-0,276205
	C6	-0,277888	C6	-0,247164	C6	-0,257993
	C7	-0,156695	C7	-0,177319	C7	-0,166580
	C8	-0,156692	C8	-0,157413	C8	-0,145802
	C9	0,072561	C9	-0,038441	C9	0,011632
	C10	0,315050	C10	0,328847	C10	0,250020

	C11	-0,104363	C11	-0,074427	C11	-0,092019
	C12	-0,104365	C12	-0,165474	C12	-0,126682
	N1	-0,085514	N1	-0,084316	N1	-0,084446
	N2	-0,085515	N2	-0,084114	N2	-0,084824
	N3	-0,251769	N3	-0,243675	N3	-0,235722
	N4	-0,251790	N4	-0,016930	N4	-0,028158
	-	-	C13	0,084517	C13	-0,016391
	-	-	C14	-0,144001	C14	0,081983
	-	-	C15	-0,177967	C15	-0,155742
	-	-	C16	-0,162385	C16	-0,145272
	-	-	C17	-0,151828	C17	-0,093599
	-	-	C18	0,112023	C18	-0,110852
	-	-	N5	0,431526	N5	0,459160
	-	-	O1	-0,331159	O1	-0,310155
	-	-	O2	-0,332019	O2	-0,328836
	C1	-0,253786	C1	-0,255387	C1	-0,271823
	C2	-0,253784	C2	-0,253121	C2	-0,270732
	C3	-0,008101	C3	0,001927	C3	-0,012610
	C4	0,168252	C4	0,168825	C4	0,154072
	C5	-0,270494	C5	-0,264203	C5	-0,271031
	C6	-0,270499	C6	-0,242855	C6	-0,243517
	C7	-0,139605	C7	-0,169386	C7	-0,139308
	C8	-0,139608	C8	-0,150902	C8	-0,172797
	C9	-0,018191	C9	-0,092319	C9	-0,050879
	C10	0,372496	C10	0,392713	C10	0,249880
	C11	-0,115644	C11	-0,093056	C11	-0,093354
	C12	-0,115645	C12	-0,186209	C12	-0,124176
Mulliken charges S_1	N1	-0,077932	N1	-0,068803	N1	-0,065991
	N2	-0,077932	N2	-0,068996	N2	-0,066460
	N3	-0,267869	N3	-0,283525	N3	-0,224826
	N4	-0,267870	N4	-0,022575	N4	-0,019521
	-	-	C13	0,134177	C13	0,114862
	-	-	C14	-0,211818	C14	-0,020288
	-	-	C15	-0,189405	C15	-0,172045
	-	-	C16	-0,146036	C16	-0,111060
	-	-	C17	-0,157772	C17	-0,074768
	-	-	C18	0,088741	C18	-0,127476
	-	-	N5	0,430141	N5	0,486281
	-	-	O1	-0,344815	O1	-0,334921
	-	-	O2	-0,346138	O2	-0,354944

Table S4. Charges for each of the atoms in compounds **(1)**, **(2)** and **(3)**, in their excited S_1 and ground S_0 states.

2. Antibacterial Activity

2.1. Preparation of inoculums

2.1.1. Culture media

Prepared by a mixture of Luria-Bertani LB broth base (7.5 g) with agar-agar (10 g), place in a clean 1-liter flask, and add 500ml of distilled water. Note that containers used for media must have vented tops and should be capable of holding 20 % more than the intended volume of medium, to allow for expansion during sterilization. Swirl the flaks to mix the powder mixture into the water as homogenously as possible. Cover the flask with aluminum foil and put it in the autoclave, set the equipment at 1atm and 120 °C for 30 minutes. Once the sterilization finishes, take the flask out and let it cool down for 40 to 60 minutes.

Set the needed petri dishes in a laminar flow hood and pour the culture media inside the petri dishes until the whole area of the plate is completely covered. Allow the plates to cool and the agar to completely solidifies.

2.1.2. Bacteria strains

Escherichia coli (DH5-alpha) was chosen for antibacterial activity evaluation. The bacterial stock cultures were incubated for 24 hours at 37 °C on Luria Bertani (LB) broth base, following by refrigeration storage at 4 °C.

2.2. Antibacterial tests

Two techniques were used to test the antibacterial activity of synthesized compounds **1, 2, b** and **c**. The agar diffusion technique and optical density (OD) measurements were applied in order to study of antibiotic efficacy of the synthesized molecules.

2.2.1. Agar diffusion

Different dilutions of the selected compounds **1**, **2**, **b** and **c** were prepared at different concentrations (1mg/ml; 0.1mg/ml and 0.01mg/ml) each one, using 5:5 water/DMSO system as solvent. Once the solutions are prepared, it was necessary to inoculate the agar plates containing the appropriate medium with the bacteria strains. Then, three µl of each solution were added onto the agar plates and incubated at 37°C for 24 hours. After that, the inhibition zones were observed under uv-light. Ampicillin (100 µg/ml), Kanamycin sulfate (100µg/ml) and the solvent system (5:5 water/DMSO) were used as positive controls.

The results obtained for the agar diffusion technique, exhibited inhibition zones for all the tested molecules. For this test the labels R11, R12, R13 corresponds to dilutions of **b** (1000 µg/ml; 100 µg/ml and 10 µg/ml), respectively. In the same way the labels R21, R22, R23 correspond to the same dilutions of **c**, R31, R32, R33 correspond to the respective dilutions of **1** and R41, R42, R43 correspond to the respective dilutions of **2**. Also, the letters K, S and A states for kanamycin, the solvent system and ampicillin, respectively (**Figure S3**).

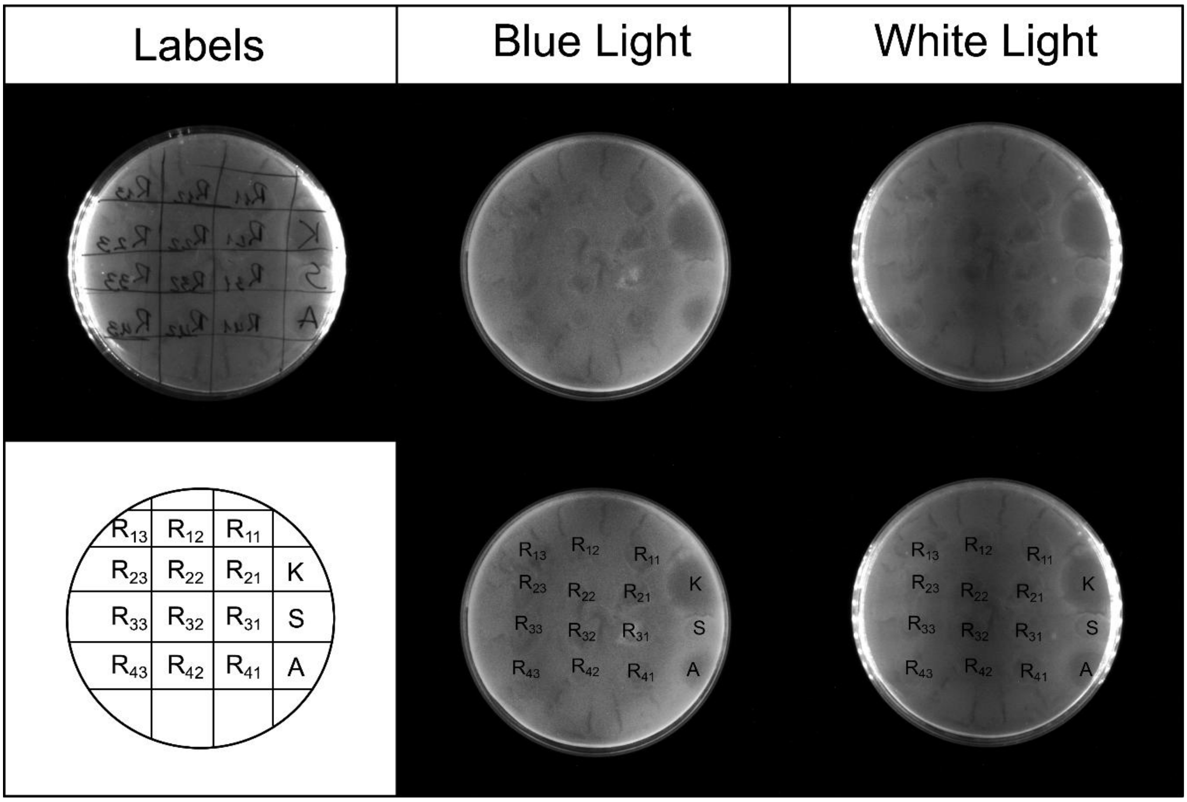

Figure S3. Antibacterial activity test performed in E. coli strains for compounds **(1, 2, b** and **c)**. Revealed under blue light and white light.

2.2.2. Optical density measurements (OD)

Optical density measurements were performed on a Thermo-scientific Nanodrop UV-Vis spectrophotometer. Absorbance measurements were taken every 30 minutes at 600nm wavelength and room temperature. Five different test tubes were prepared with 2.5ml of Luria Bertani (LB) broth base and 135μL of *E. coli* (DH5-alpha) strains to obtain an initial OD_{600} of 0,02. For tube 1 nothing else was added, while for tube 2 it was additionally added 100 μL of the solvent system (5:5; water/DMSO). For tubes 3, 4, 5 and 6 it was added 100μL of 1mg/ml solutions of compounds **b**, **1**, **2** and **c** respectively. **(Table S5)** All test tubes were kept in a shaker incubator at 37°C for the entire measurement period.

**T. tube**	**1**	**2**	**3**	**4**	**5**	**6**
LB Broth	2.5 ml	2.5ml	2.5ml	2.5ml	2.5ml	2.5ml
E. coli strain	135 ul	135 ul	135 ul	135 ul	135 ul	135 ul
Water	-	50 ul	50 ul	50 ul	50 ul	50 ul
DMSO	-	50 ul	50 ul	50 ul	50 ul	50 ul
Compound b	-	-	100 ug	-	-	-
Compound 1	-	-	-	100 ug	-	-
Compound 2	-	-	-	-	100 ug	-
Compound c						100 ug

Table S5. Content of each test tube for the OD_{600} measurements.

As mentioned in the main paper section compounds **1** and **2** showed a reduction of 4.7 and 4.4 %, respectively. Whereas, **c** reaches an inhibition capacity of 7.9% and **b** reaches the highest inhibition capacity, with a value of 14.3%. Thus, taking into account the chemical structures for each of the studied compounds and their respective inhibition capacity it is possible that the

cyano the primary and secondary amine groups played an important role for the antibacterial capacity in each of the molecules. This is because the inhibition activity reduces with the number of cyano groups present in each of the molecules, like compound **b** that has the most important inhibition activity of all the compounds but it strongly reduces for **1** and **2**. Also, the primary and secondary amines seem to play an important role because as the primary or secondary amines get oxidized to secondary or tertiary amines respectively the antibacterial activity also reduces, like in the case of compound **c** in comparison with **1** and **2**. Finally, comparing the antibacterial activities of **1** and **2** it seems that the presence of the nitro-benzene moiety does not contribute to the biological activity. However, it is necessary to perform more investigations to obtain a further insight into the possible conformational changes that could affect the antibacterial activity of the DADQs derivative.

3. FT-IR Spectrums

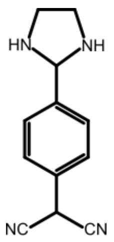

1

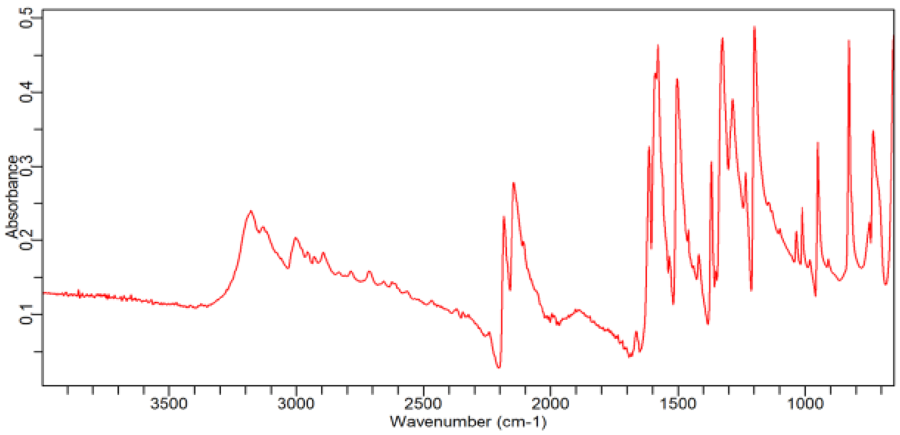

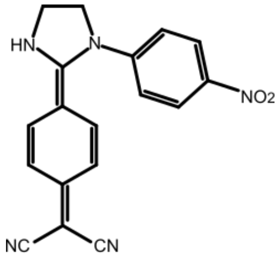

2

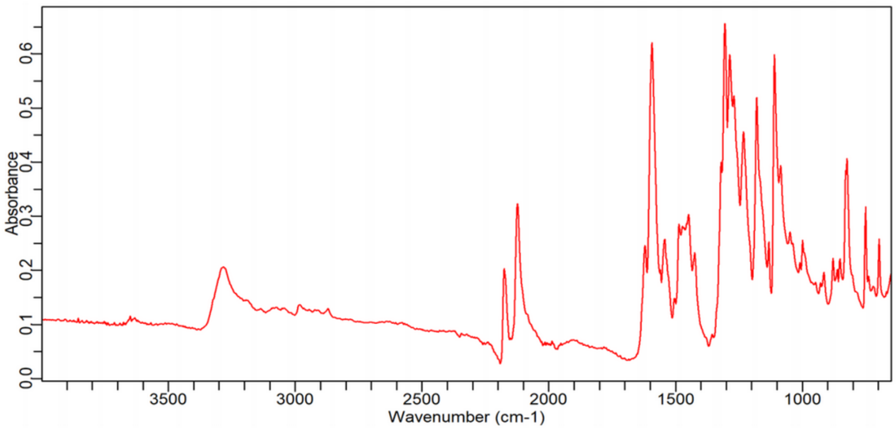

4. ESI-MS

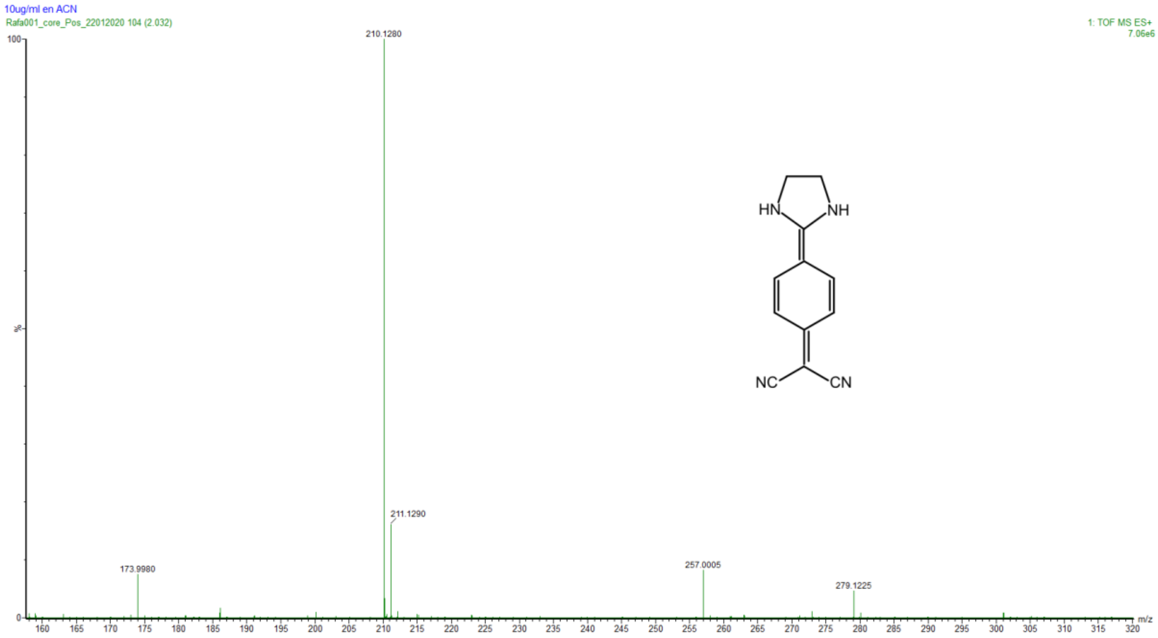

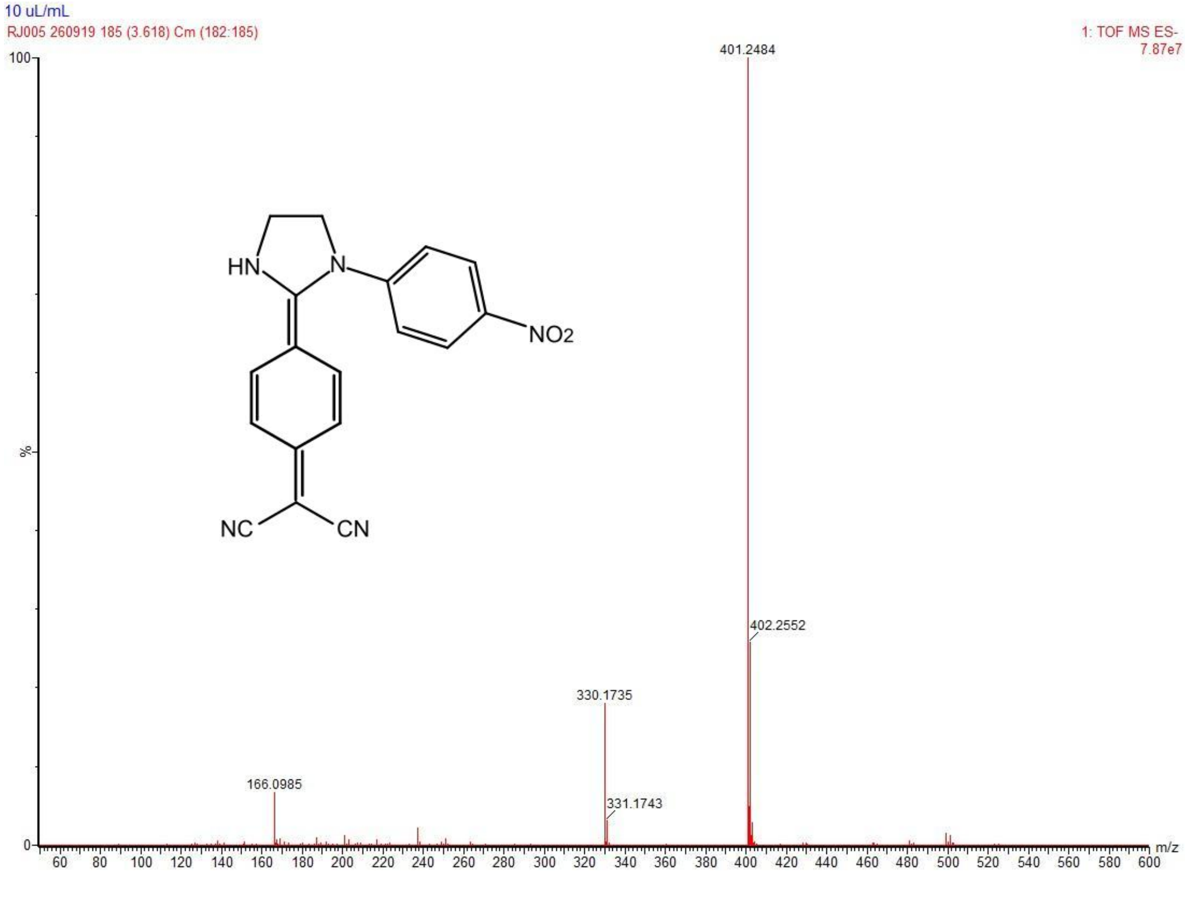

5. Optimized structures coordinates

5.1. Compound 1

5.1.1. Ground state S0

26
Compound 1_S0
C -0.3415820000 1.1980660000 0.0821000000

C	1.0274200000	1.2004700000	0.0865590000
C	-1.0609690000	0.0000010000	-0.0000310000
C	-0.3415900000	-1.1980690000	-0.0821570000
C	1.7626000000	-0.0000090000	-0.0000340000
C	1.0274110000	-1.2004840000	-0.0866250000
H	-0.8610910000	2.1425970000	0.1737880000
H	1.5541030000	2.1414800000	0.1642190000
H	1.5540880000	-2.1414970000	-0.1642890000
H	-0.8611140000	-2.1425920000	-0.1738480000
C	-2.4978820000	-0.0000040000	-0.0000220000
C	3.1918700000	-0.0000080000	-0.0000400000
C	3.9193790000	1.1957490000	0.0798730000
C	3.9193660000	-1.1957720000	-0.0799310000
N	-3.2575250000	1.0623950000	-0.2267310000
N	-3.2575050000	-1.0624310000	0.2266820000
C	-4.6740580000	0.7053110000	-0.3011040000
C	-4.6740320000	-0.7053250000	0.3012090000
H	-5.0013490000	0.7040030000	-1.3404680000
H	-5.2848070000	1.4032640000	0.2637080000
H	-5.2848480000	-1.4032700000	-0.2635380000
H	-5.0012090000	-0.7040120000	1.3406090000
N	4.5024250000	-2.1907240000	-0.1461580000
N	4.5022050000	2.1908220000	0.1463440000
H	-2.8943030000	1.9035830000	-0.6408900000
H	-2.8942530000	-1.9035440000	0.6409710000

5.1.2. Excited state S1

26
Compound 1_S1 Excited

C	-0.3415590000	1.2217430000	0.0549930000
C	1.0199770000	1.2145650000	0.0533490000
C	-1.0800100000	0.0000020000	-0.0000460000
C	-0.3415580000	-1.2217360000	-0.0551260000
C	1.7525860000	0.0000080000	-0.0001320000
C	1.0199780000	-1.2145530000	-0.0535580000
H	-0.8613560000	2.1664110000	0.1246140000
H	1.5552150000	2.1536410000	0.0994350000
H	1.5552180000	-2.1536270000	-0.0996770000
H	-0.8613550000	-2.1664060000	-0.1247190000
C	-2.4860530000	-0.0000010000	0.0000010000
C	3.1846610000	0.0000100000	-0.0001780000
C	3.9268710000	1.1914720000	0.0602320000
C	3.9268720000	-1.1914660000	-0.0602880000
N	-3.2757120000	1.1086750000	-0.0622070000
N	-3.2757040000	-1.1086800000	0.0622620000
C	-4.6623300000	0.7065320000	-0.2863860000
C	-4.6623080000	-0.7065400000	0.2865360000
H	-4.8947380000	0.6987470000	-1.3545940000
H	-5.3569970000	1.3693220000	0.2228190000
H	-5.3570080000	-1.3693330000	-0.2226230000
H	-4.8946430000	-0.6987560000	1.3547590000

N	4.5207420000	-2.1802980000	-0.1082490000
N	4.5207500000	2.1802740000	0.1087030000
H	-2.9288380000	1.9305340000	-0.5309330000
H	-2.9287960000	-1.9305340000	0.5309720000

5.2. Compound 2

5.2.1. Ground State S0

38

Compound 2_S0_NO2para

C	-2.1554510000	1.6771620000	0.6833200000
C	-3.2026390000	0.7949060000	0.6689760000
C	-1.0018150000	1.4370280000	-0.0726130000
C	-3.1653550000	-0.3723140000	-0.1215190000
C	-2.0041300000	-0.5925600000	-0.8926180000
C	-0.9521010000	0.2815240000	-0.8620760000
H	-2.2146950000	2.5513270000	1.3185700000
H	-4.0718340000	0.9959580000	1.2796190000
H	-1.9449800000	-1.4673010000	-1.5252700000
H	-0.0875050000	0.0826100000	-1.4791330000
C	0.0767750000	2.3888170000	-0.0677910000
N	-0.1022790000	3.6901310000	0.0392200000
N	1.3813160000	2.1162540000	-0.2062120000
C	1.1556380000	4.4298810000	0.0759840000
C	2.1462690000	3.3648500000	-0.3963490000
H	1.3619100000	4.7710800000	1.0897710000
H	1.1174260000	5.2859060000	-0.5899630000
H	3.0533800000	3.3378270000	0.1989540000
H	2.4061410000	3.4852650000	-1.4471200000
C	2.0208890000	0.8683560000	-0.0587680000
C	3.0620240000	0.5446690000	-0.9180770000
C	3.7228720000	-0.6578170000	-0.7754060000
C	1.6517980000	-0.0008010000	0.9615360000
C	2.3001200000	-1.2079090000	1.1007370000
C	3.3259990000	-1.5196710000	0.2275820000
H	3.3502770000	1.2242870000	-1.7063960000
H	0.8623610000	0.2688580000	1.6463250000
H	2.0276960000	-1.8958740000	1.8853020000
H	4.5290110000	-0.9300920000	-1.4379490000
C	-4.2579300000	-1.2924810000	-0.1429840000
C	-5.4139170000	-1.0665740000	0.6177390000
C	-4.2164370000	-2.4536410000	-0.9278490000
N	-6.3583330000	-0.8627820000	1.2506880000
N	-4.1657840000	-3.4047840000	-1.5811790000
N	4.0194220000	-2.7978510000	0.3777930000
O	3.6448650000	-3.5513690000	1.2535790000
O	4.9357810000	-3.0435280000	-0.3806960000
H	-1.0104020000	4.1219850000	0.0231060000

5.2.2. Excited State S1

38

Compound 2_S1 Excited_NO2para

C	2.2421730000	1.6684270000	-0.6748460000
C	3.3028450000	0.8140340000	-0.6212220000
C	1.0298530000	1.3683500000	-0.0024660000
C	3.2321920000	-0.3969310000	0.1121250000
C	2.0271800000	-0.6840140000	0.8005810000
C	0.9633660000	0.1632830000	0.7475680000
H	2.3156160000	2.5710280000	-1.2642530000
H	4.2106220000	1.0591970000	-1.1551720000
H	1.9543940000	-1.5900550000	1.3862890000
H	0.0721140000	-0.0739090000	1.3070890000
C	-0.0401900000	2.3043730000	-0.0338430000
N	0.1377230000	3.6306930000	-0.2850300000
N	-1.3435970000	2.0638430000	0.2685400000
C	-1.1114720000	4.3625180000	-0.0706490000
C	-2.0024760000	3.3363260000	0.6248320000
H	-1.5305530000	4.6773330000	-1.0263810000
H	-0.9436020000	5.2414850000	0.5444590000
H	-3.0229270000	3.3456070000	0.2565260000
H	-2.0062330000	3.4546410000	1.7084460000
C	-2.0477420000	0.8957080000	0.0826050000
C	-3.2243880000	0.6719550000	0.8178320000
C	-3.9204740000	-0.4981350000	0.6705070000
C	-1.6176190000	-0.0762760000	-0.8368460000
C	-2.3145130000	-1.2466770000	-0.9822560000
C	-3.4589770000	-1.4650750000	-0.2214330000
H	-3.5690200000	1.4097380000	1.5270750000
H	-0.7533220000	0.1115120000	-1.4544090000
H	-1.9967670000	-1.9902800000	-1.6960970000
H	-4.8144410000	-0.6834840000	1.2445410000
C	4.3333860000	-1.2925250000	0.1542670000
C	5.5385860000	-1.0196650000	-0.5202450000
C	4.2765450000	-2.5019240000	0.8719610000
N	6.5199690000	-0.7844130000	-1.0768950000
N	4.2157610000	-3.4895730000	1.4630000000
N	-4.1797090000	-2.6911370000	-0.3690110000
O	-3.7473090000	-3.5389420000	-1.1431620000
O	-5.2059880000	-2.8513140000	0.2839040000
H	1.0098070000	4.0608270000	-0.0191170000

5.3. Compound 3

5.3.1. Ground State S0

38
Compound 3_S0_NO2orto

C	-0.5606560000	0.1315490000	-1.1065700000
C	-1.8651170000	0.5361120000	-1.0159350000
C	-0.1365430000	-1.0633580000	-0.5116650000
C	-2.8230360000	-0.2213330000	-0.3103790000
C	-2.3835310000	-1.4224920000	0.2839020000
C	-1.0822880000	-1.8333290000	0.1775830000

H	0.1334870000	0.7355560000	-1.6723280000
H	-2.1660910000	1.4548380000	-1.4998260000
H	-3.0847450000	-2.0276380000	0.8415200000
H	-0.7792080000	-2.7480140000	0.6696650000
C	1.2248060000	-1.5221100000	-0.6029060000
C	-4.1806420000	0.2102010000	-0.2020980000
N	1.5663960000	-2.7954240000	-0.6049150000
N	2.3108760000	-0.7485020000	-0.6804980000
C	-4.6118270000	1.4090190000	-0.7875560000
C	-5.1318740000	-0.5481570000	0.4949800000
N	-5.9021350000	-1.1857660000	1.0731550000
N	-4.9493900000	2.3996020000	-1.2763700000
C	3.5384080000	-1.5658910000	-0.6422310000
C	3.0010490000	-2.9926860000	-0.7896320000
H	4.0286110000	-1.4086160000	0.3169140000
H	4.2136310000	-1.2814600000	-1.4437740000
H	3.4015210000	-3.6682680000	-0.0392020000
H	3.1919050000	-3.4043360000	-1.7790030000
C	2.3746810000	0.6301630000	-0.3618690000
C	1.9476960000	1.1546830000	0.8559120000
C	1.9825790000	2.5111980000	1.1088690000
C	2.4976050000	3.3663300000	0.1536340000
C	2.9542650000	2.8591430000	-1.0502330000
C	2.8813740000	1.5015360000	-1.3084690000
H	3.1974860000	1.1040320000	-2.2626740000
H	2.5418160000	4.4271600000	0.3529850000
H	3.3545720000	3.5235730000	-1.8027410000
H	1.6231350000	2.8801220000	2.0573840000
H	0.8932620000	-3.5264320000	-0.7634770000
N	1.4537010000	0.2910000000	1.9290070000
O	0.5817410000	0.7282610000	2.6464280000
O	1.9598570000	-0.8070560000	2.0573080000

5.3.2. Excited State S1

38
Compound 3_S1 Excited_NO2orto

C	-0.2903270000	-0.0867610000	-1.4340530000
C	-1.6045410000	0.2556490000	-1.3512040000
C	0.1937960000	-1.1905510000	-0.7185400000
C	-2.4918970000	-0.4997740000	-0.5402960000
C	-2.0036470000	-1.6756830000	0.0887970000
C	-0.6896910000	-2.0139760000	-0.0101000000
H	0.3851930000	0.5144760000	-2.0232140000
H	-1.9714630000	1.1221090000	-1.8813970000
H	-2.6742820000	-2.2812160000	0.6804760000
H	-0.3103600000	-2.8676850000	0.5301900000
C	1.6271130000	-1.4315040000	-0.5996300000
C	-3.8058970000	-0.0745810000	-0.3332830000
N	2.1808160000	-2.6254300000	-0.6249470000
N	2.5219040000	-0.4736730000	-0.4379960000
C	-4.3064620000	1.1101040000	-0.9239750000

C	-4.7037880000	-0.7978060000	0.4875710000
N	-5.4274460000	-1.3853960000	1.1574520000
N	-4.7049970000	2.0742760000	-1.4028090000
C	3.8347590000	-1.0752300000	-0.1478800000
C	3.6400250000	-2.5306880000	-0.5797510000
H	4.0126760000	-0.9838900000	0.9233090000
H	4.6242680000	-0.5691380000	-0.6942380000
H	4.0564090000	-3.2400450000	0.1289470000
H	4.0521480000	-2.7248820000	-1.5691170000
C	2.2221530000	0.8710820000	-0.0811220000
C	1.2876430000	1.2038590000	0.9171780000
C	0.9313950000	2.5496610000	1.0597110000
C	1.5384230000	3.5265310000	0.3055510000
C	2.5112370000	3.1974970000	-0.6340000000
C	2.8311190000	1.8693090000	-0.8255760000
H	3.5370530000	1.5819000000	-1.5933080000
H	1.2566650000	4.5604860000	0.4524340000
H	2.9912090000	3.9633820000	-1.2254100000
H	0.1843410000	2.7934210000	1.7979990000
H	1.6871340000	-3.4479410000	-0.9295060000
N	0.6895400000	0.2560160000	1.7475530000
O	-0.3978920000	0.5498360000	2.3466730000
O	1.2756180000	-0.8728790000	1.9347850000

References

1. [1] B. L. Fierz-david Hans Eduard, *Fundamental processes of dye chemistry*, Preface to. London: Interscience publishers, 1949.
2. [2] "https://www.sigmaaldrich.com/catalog/product/aldrich/c59122?lang=en&region=EC." .
3. [3] P. Rietsch *et al.*, "Diaminodicyanoquinones: Fluorescent Dyes with High Dipole Moments and Electron-Acceptor Properties," *Angew. Chemie - Int. Ed.*, vol. 58, no. 24, pp. 8235–8239, 2019, doi: 10.1002/anie.201903204.
4. [4] G. Glotz, R. Lebl, D. Dallinger, and C. O. Kappe, "Integration of Bromine and Cyanogen Bromide Generators for the Continuous-Flow Synthesis of Cyclic Guanidines," *Angew. Chemie - Int. Ed.*, vol. 56, no. 44, pp. 13786–13789, 2017, doi: 10.1002/anie.201708533.
5. [5] T. M. Wróbel *et al.*, "Synthesis, structural studies and molecular modelling of a novel imidazoline derivative with antifungal activity," *Molecules*, vol. 20, no. 8, pp. 14761–14776, 2015, doi: 10.3390/molecules200814761.
6. [6] M. Allali, E. Benoist, N. Habbadi, M. Gressier, A. Souizi, and M. Dartiguenave, "Design and synthesis of new ethylenediamine or propylenediamine diacetic acid derivatives for Re(I) organometallic chemistry," *Tetrahedron*, vol. 60, no. 5, pp. 1167–1174, 2004, doi: 10.1016/j.tet.2003.11.058.
7. [7] P. Srujana, T. Gera, and T. P. Radhakrishnan, "Fluorescence enhancement in crystals tuned by a molecular torsion angle: A model to analyze structural impact," *J. Mater. Chem. C*, vol. 4, no. 27, pp. 6510–6515, 2016, doi: 10.1039/c6tc01610c.
8. [8] A. T. Rhys Williams, S. A. Winfield, and J. N. Miller, "Relative fluorescence quantum yields using a Computer-controlled luminescence spectrometer," *Analyst*, vol. 108, no. 1290, pp. 1067–1071, 1983, doi: 10.1039/an9830801067.
9. [9] Horiba, "A Guide to Recording Fluorescence Quantum Yields Introduction:" Horiba, London, [Online]. Available: https://static.horiba.com/fileadmin/Horiba/Application/Materials/Material_Research/Quantum_Dots/quantumyieldstrad.pdf.
10. [10] D. B. Siano and D. E. Metzler, "Band shapes of the electronic spectra of complex molecules," *J. Chem. Phys.*, vol. 51, no. 5, pp. 1856–1861, 1969, doi: 10.1063/1.1672270.
